# 

**Published:** 1878-03

**Authors:** 


### Books and Pamphlets Received.

Ziemssen's Cyclopœdia, Vol. XIV. Diseases of the Nervous System. New York, Wm. Wood & Co. 1877; Buffalo, J. H. Matteson.

The Science and Art of Surgery by John Eric Erichsen F. R. S., F. R. C. S. Two Vol. Philadelphia, Henry C. Lea 1878. Buffalo, T. H. Butler.

Transactions of the American Medical Association. Vol. 28. 1877.

Transactions of the Medical Society of the State of New York. 1877.

Mortuary Statistics of the Mutual Life Ins. Co. of N. Y. Part 2.

Practical Gynæcology, by Heywood Smith M. A. M. D. Oxon. Philadelphia, Lindsay and Blakiston. 1878. Buffalo, T. H. Butler.

Handbook of the Practice of Medicine by M. Charteris, M. D. Philadelphia, Lindsay and Blakiston. 1878. Buffalo, T. H. Butler.

State Regulations of Vice by Aaron M. Powell. New York, Wood and Holbrook. 1878.

Bintz's Elements of Therapeutics. Translated by Edward I. Sparks M. A., M. B. Oxon., New York, Wm. Wood & Co. 1878. Buffalo, H. H. Otis.

Transactions of the Ohio Medical Society, 1877.

Address before the Rocky Mountain Medical Association, by J. M. Toner M. D.

Circular No. 10. Approved Plans and Specifications for Post Hospitals.

Studies in Pathological Anatomy by Francis Delafield M. D. No. 1. February 1878. New York, Wm. Wood & Co.

Landmarks Medical and Surgical by Luther Holden F. R. C. S. Philadelphia, Henry C. Lea. 1878. Buffalo, T. H. Butler.

Malaria and Struma in their relation to the Etiology of Skin Diseases, by L. P. Yandell, Jr., M. D. Louisville, Ky. Reprint from American Practitioner January, 1878.

### Contents of March Number.

### ORIGINAL.

ART. I.—Clinical Lecture on Club-Foot. By Prof. J. F. Miner, M. D. 281

### CORRESPONDENCE.

**THE BUFFALO**

### Medical and Surgical Journal

**PUBLISHED MONTHLY,**

Containing Original Articles, Reports of Medical Societies and Hospitals, Editorial, Reviews, Correspondence, Army News, etc., etc ; including the usual variety of Medical Periodical Publications. Specimen copies sent on application.

Address Buffalo Medical & Surgical Journal, Buffalo, N. Y.

TERMS OF SUBSCRIPTION, $3.00 PER YEAR, IN ADVANCE.

All communications for publication shoud be sent before the twentieth (20th) of each month, or they will of necessity lie over until the next number. No change can be made in advertisements after the twenty-fifth. Address, Buffalo Medical and Surgical Journal, 978 Main Street.

Correspondence and original articles are solicited. Publication is no evidence that the views of the author are endorsed.

